# Strain Analysis of Ti6Al4V Titanium Alloy Samples Using Digital Image Correlation

**DOI:** 10.3390/ma13153398

**Published:** 2020-07-31

**Authors:** Karolina Karolewska, Bogdan Ligaj, Dariusz Boroński

**Affiliations:** Faculty of Mechanical Engineering, UTP University of Science and Technology in Bydgoszcz, al. Prof. S. Kaliskiego 7, 85-796 Bydgoszcz, Poland; bogdan.ligaj@utp.edu.pl (B.L.); dariusz.boronski@utp.edu.pl (D.B.)

**Keywords:** additive manufacturing, DMLS technology, titanium alloy Ti6Al4V, HV10 hardness, digital image correlation (DIC)

## Abstract

Digital image correlation (DIC) is a non-contact optical method that allows measuring displacements on a plane used to determine the strains caused by external loads of a structural element (mechanical or thermal). Currently, digital image correlation is a widely used experimental technique to assess the mechanical behavior of materials, in particular cracking characteristics and destruction methods of various structural elements. In this paper, the DIC method is applied to determine local strains of titanium alloy Ti6Al4V specimen. The samples used in the tests were made with two different technologies: (a) from a drawn bar by machining process; and (b) by the additive manufacturing method Direct Metal Laser Sintering (DMLS). The aim of the paper is to present the mechanical properties test results of the Ti6Al4V titanium alloy produced by the DMLS additive manufacturing under static loads using the digital image correlation method. As a result of the tests carried out on the drawn bar specimens, it was concluded that the change in the measurement base affects the difference in the Young’s E modulus value in the range from 89.2 to 103.8 GPa. However, for samples formed using the DMLS method, the change in the Young’s modulus value was from 112.9 to 115.3 GPa for the same measurement base.

## 1. Introduction

Additive technologies are increasingly used in the structural elements production due to a number of advantages, i.e., short production cycle, high efficiency, and production flexibility [1]. Additive manufacturing has contributed to the widespread use of some metal alloys that are difficult to produce with conventional machining methods. Titanium alloy belongs to this materials group [2,3]. The Ti6Al4V titanium alloy is the most commonly applied material due to its excellent mechanical properties. In the production of components made using Ti6Al4V alloy powder the following additive manufacturing technologies are frequently adopted [4,5]: Laser Melting Deposition (LMD), Selective Laser Melting (SLM), and Direct Metal Laser Sintering (DMLS). These methods use a laser as an energy source to melt the metal powder. Laser energy, powder movement, and dynamic interaction between them have a significant impact on the printed material structure and its mechanical properties [4].

Chang et al. [6] presented the research results of titanium alloy Ti6Al4V samples made by the means of the SLM additive technology. The annealing treatment, which was performed favorably, affected the tensile strength (S_u_ = 953 MPa) and the elongation (A = 17.7%).

Bebei et al. [7] presented the static tests results and the microstructure characteristics of the Ti6Al4V material manufactured by the SLM method. The elements for testing were printed horizontally on the EOS M290. The printout was characterized by the following parameters: scanning speed 1200 mm/s, laser power 280 W, and layer thickness 30 μm. The material was subjected to a static tensile test, which resulted in tensile strength of S_u_ = 1261 MPa and elongation of A = 10.2%. The tensile strength of the Ti6Al4V alloy parts that were produced by selective laser melting was very similar to the strength of forged elements from the same alloy, although the elongation was slightly lower. Excellent mechanical properties in terms of tensile strength and plasticity for parts manufactured by laser melting method were attributed to almost full density.

Sun et al. [8] gave the results of material print direction impact on strength under static and variable loads of the titanium alloy Ti6Al4V produced by the SLM method. Specimen print parameters were: laser power 350W, laser beam diameter 0.08 mm, scanning speed 1000 mm/s, and layer thickness 0.06 mm. The samples were manufactured in three directions: 0°, 45°, and 90°. As-built specimens were annealed for 5 h at 750–850 ℃, and then cooled under argon to room temperature. The material different mechanical parameters were obtained for each direction. For the 0° specimens, the following values were gained: E = 111.55 GPa, S_u_ = 935 MPa, and S_y_ = 857 MPa. For the 45° samples, E = 113.26 GPa, S_u_ = 963 MPa, and S_y_ = 883 MPa. For the 90° samples, E = 115.87 GPa, S_u_ = 953 MPa, and S_y_ = 888 MPa. 

Liang et al. [9] presented research results concerning the various heat treatment methods impact on the material structure of samples produced by the SLM method. The material heat treatment was carried out at heightened temperature (600, 800, 850, and 900 °C) for 4 h and cooled in air to room temperature. The material mechanical properties values were obtained for specimens without heat treatment: S_u_ ≈ 1230 MPa, S_y_ ≈ 1150 MPa, and A ≈ 8.5%. The following results were obtained for specimens after heat treatment at 600 °C: S_u_ ≈ 1210 MPa, S_y_ ≈ 1180 MPa, and A ≈ 8.5%. For samples after heat treatment at 800 °C, the results were: S_u_ ≈ 1080 MPa, S_y_ ≈ 990 MPa, and A ≈ 12,1%. For samples after heat treatment at 850 ℃, the results were: S_u_ ≈ 1040 MPa, S_y_ ≈ 970 MPa, and A ≈ 12.3%. For samples after heat treatment at 900 °C, the results were: S_u_ ≈ 990 MPa, S_y_ ≈ 900 MPa, and A ≈ 11.5%.

Karolewska et al. [10] gave the research results of Ti6Al4V titanium alloy according to [11] standard. The test specimens were made using the DMLS method, and their diameter was 6 mm. The geometrical dimensions of the samples were adopted according to [12] standard. The printing process was characterized by the following parameters: laser power 200 W, minimum layer thickness 30 μm, and scanning speed up to 7 m/s. After the manufacturing process, the specimens were annealed. Based on testing under static load, the following strength parameters were achieved: E = 114.5 GPa, S_u_ = 1127 MPa, S_y 0.2_ = 1052 MPa, and A = 15.5%.

Benedetti et al. [13] presented the static tests results for Ti6Al4V material produced by the Selective Laser Sintering (SLS) method using a 3DSystem ProX 300 printer. Two types of samples were adopted for testing. The first of these were tested in the as-built state, immediately after the printing process. The second part was subjected to hot isostatic pressing (HIP) treatment at 920 °C, aimed at removing pores and obtaining the full density of the material and modifying its microstructure. As-built specimens show a martensitic microstructure and have a higher yield strength (Sy = 1022 MPa) and tensile strength (Su = 1092 MPa) than heat-treated samples, but lower elongation in HIP condition (A = 16.5%). The properties of HIP treated samples were: elongation A = 22.5%, yield strength Sy HIP = 894.7 MPa, and tensile strength Su HIP = 962.3 MPa. The HIP heat treatment changed the parameters of the Ti6Al4V material produced by the SLS method.

Benedetti et al. [14] found that mechanical properties are closely related to the material microstructure. A static tensile test was carried out for printed and untreated samples as well as for specimens subjected to HIP hot isostatic pressing. The Young’s modulus for both types of samples assumes similar values of about 110 GPa. However, there is a larger difference in terms of yield point, strength, and total elongation. Specimens with martensitic microstructure (samples without heat treatment) show higher yield point and tensile strength, but lower elongation.

Quintana et al. [15] determined the mechanical properties made by the SLM additive method for two types of Ti6Al4V material: Grades 5 and 23. They examined the impact of a slight increase in oxygen content on the mechanical properties of SLM Ti6Al4V. The specimens’ microstructure and mechanical properties after the printing process and after HIP treatment with oxygen content were assessed corresponding to the Ti6Al4V ELI (Grade 5) ranges 0.10–0.11% and 0.16–0.17%. Ti6Al4V Grade 5 material showed higher yield point and strength than ELI type, in the case of both as-built samples and after HIP treatment. Grade 5 material after HIP treatment had greater elongation than Grade 23 type. Conversely, the elongation of the Grade 5 as-built material was lower than that of the ELI type.

Rafi et al. [16] produced specimens from Ti6Al4V and 15-5 PH materials using SLM. Their tensile strength was determined. Strength properties were compared in relation to the orientation of the manufactured element. The samples built horizontally showed relatively better tensile properties compared to the vertically built specimens. They presented that the tensile strength of Ti6Al4V produced by SLM is higher than that of hot-machined parts due to the martensitic microstructure. However, its plasticity was lower. The tensile strength of Ti64 and PH1 samples was found to be comparable or even better than the forged material. Tensile properties affect the direction of printing. Horizontal orientation is slightly better than vertical orientation in terms of tensile strength.

The test results presented in [7,13,14,15,16] indicate an increase in the tensile strength and yield point of the material produced by the additive method (in as-built state) compared to the heat-treated material.

A short strength parameter analysis of the material received from additive manufacturing technology indicates that the results obtained have a large spread in regards to the material’s tensile strength S_u_, yield point S_y_, and elongation A. Not all researchers determine and analyze changes in the Young’s modulus value. The change in strength parameters is influenced by a number of factors related to the material printing process by the additive manufacturing method, as well as to the process of formed elements heat treatment. The research methods applied to assess strength properties are the same as those adopted for testing materials produced by metallurgical processes. In the case of materials created by additive manufacturing methods, we deal with a number of layers connected together, and the weakest connection determines the entire element strength. On this assumption, the optical image digital correlation method can be selected to assess the test object’s (specimens) individual area displacement.

Digital Image Correlation (DIC) is a non-contact optical method that allows measuring displacements on a plane used to determine the strains caused by external loads of a structural element (mechanical, thermal) [17]. This method uses digital images in which the pixels are the smallest observable elements. The measurement consists of taking a series of photos before and after loading the test object. The object surface being tested must have some random texture (macular structure). One of the images in the series is selected as the reference image for all subsequent analyses [18]. The reference image is divided into small rectangular areas, which are called subsets. The algorithm tracks each subset position from the reference image in all other images of the measurement series. Finding subsets is done by calculating the mutual correlation coefficient. For each subset, displacement vectors in the plane (u and v) are calculated; using triangulations, off-plane displacements (w) are determined. Through the use of interpolation methods, sub-pixel accuracy is achieved. The output is a set of displacement maps that can then be adopted to calculate strain maps in the plane (ε_xx_, ε_yy_). In addition, various material mechanical parameters, including Young’s modulus and Poisson’s ratio, can be determined on the basis of calculated displacement or deformation fields [19]. Currently, digital image correlation is a widely used experimental technique to assess the various materials mechanical behavior, in particular cracking characteristics and destruction methods of different structural elements. Digital image correlation is also often used to observe welded joints [20].

The material mechanical properties, and above all the Young’s modulus, are of great importance due to the calculation results under static and variable loads by the means of analytical and numerical methods [21,22,23]. The vast majority of structural components are designed for the extent to which Hooke’s law applies.

The aim of the paper is to present the mechanical properties test results of the Ti6Al4V titanium alloy produced by the DMLS additive manufacturing under static loads using the digital image correlation method.

The study included conducting tests related to the mechanical properties (S_y_, S_u_, A, Z, and E) and determining the hardness of the material.

## 2. Experimental Studies

### 2.1. Research Object

The study used titanium alloy Ti6Al4V, which belongs to two-phase alloys. The European standard ISO 5832 [11] specifies the percentage of alloying elements contained in the alloy (Table 1).

In this research, samples manufactured by two technologies were adopted:

a) as a result of turning a drawn bar with a diameter of 12 mm, the material was annealed; and
b) by the use of additive technology (DMLS).

**Figure 1:**
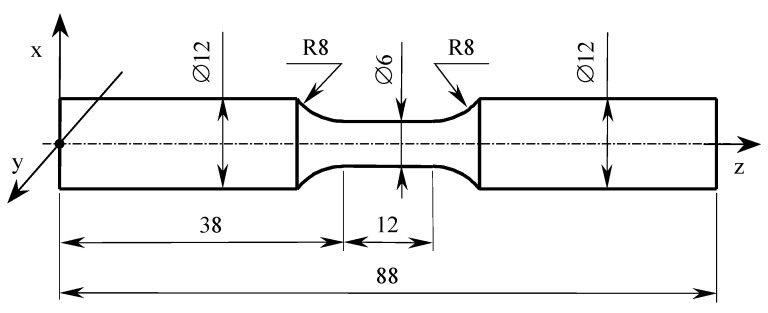
Specimen geometric features in mm for strength tests.

Ti6Al4V is a two-phase alloy consisting of α and β phases. Drawn bar (DB) samples were made of 1-m drawn rods with a diameter of 12 mm. The aim of drawing process is to obtain products in the form of bars or wires characterized by very precise cross-sectional dimensions, a smooth, bright surface, and specific mechanical properties that can only be obtained with this production method. As a result of drawing, the geometric and mechanical properties of the material change, the transverse dimensions are reduced, and the length increases, without changing the volume. As a result of plastic deformation in the die, the material also becomes hardened—the strength properties increase and the plastic properties decrease. Through the rolling process, samples were obtained as shown in Figure 1. Then, they were subjected to an annealing process at the temperature of 840 °C for 1 h and cooled in a furnace to ambient temperature. The use of annealing treatment is to obtain a stable structure of the material.

**Figure 2:**
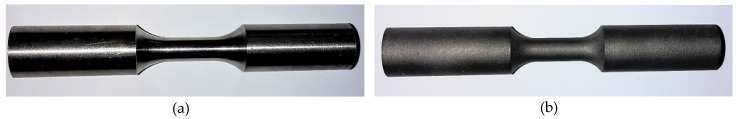
Specimen for strength tests: (**a**) drawn bar turning; and (**b**) manufactured by DMLS.

The test sample geometrical form was determined on the basis of the European standard [24]. The specimen dimensions are shown in Figure 1, while the physical form is presented in Figure 2.

Two types of samples for strength tests were made. Their physical form is given in Figure 2. 

### 2.2. Production of Specimens by the Additive Manufacturing Method

A common feature of each of the additive technologies is the ability to produce a structural element of any geometry, for which production using other manufacturing techniques would be complicated or even impossible. Over the years, many additive technologies have been created and developed using various materials types in the printing process—from polymer materials to metals. One of the printing technologies in metal is the DMLS method.

**Figure 3:**
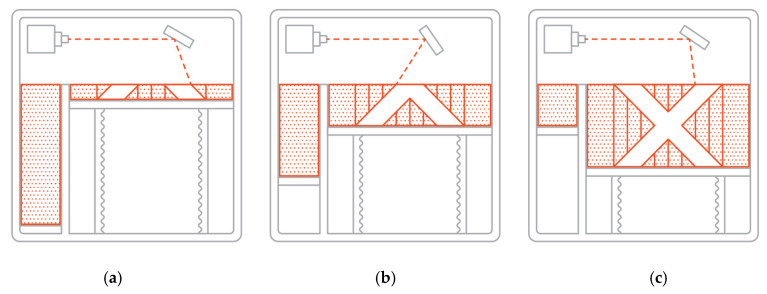
Schematic presentation of the element manufacturing process by the DMLS method [25]: (**a**) initial stage; (**b**) in the middle of the element production; and (**c**) final stage.

It is a selective laser sintering method based on metallic powder layers sintering by means of a laser beam. The samples manufacturing process by DMLS method is presented as a scheme in Figure 3. A detailed description of the method is given in [25]. Most producers of devices for components using additive manufacturing introduce their own technology designations onto the market, reserving the right to use them exclusively. In the case of the EOS company, on whose printer our samples were produced, describes its technology as DMLS. As the name suggests, DMLS produces an element by sintering individual layers, while the SLM technology completely melts these layers. Sintering processes do not fully melt the powder, but warm it up to the point where its particles can fuse together at the molecular level. With laser melting, we can get fully melt powder into a homogeneous part.

Specimens for strength tests were made using the EOS M280 machine (Materialise, Leuven, Belgium) with the dimensions of the working platform of 250 mm × 250 mm × 325 mm. The printing process was characterized by the following parameters: laser power 200 W, minimum layer thickness 30 μm, and scanning speed up to 7 m/s. The sample print direction was consistent with the *z*-axis (Figure 1). Titanium powder with the following parameters was used: grain size: 53–105 μm; grain shape: spherical; grain size distribution: D50 (72 μm); internal friction angle: ≤ 40°; grain sphericity: Φ ≥ 0.95; and bulk density: 2.56 g/cm^3^. In [10], the method for producing samples used in the tests is described in detail. During one technological process, a specimens group adopted to the tests under various load conditions was produced. The results obtained in [10] and included below were achieved under the same conditions.

### 2.3. Measurement Results of Specimens Geometric Features

#### 2.3.1. Offset Radius and Working Part Length Measurement

The measurements of selected geometrical features, i.e., offset radius values and the working part length, were carried out on a Mitutoyo Formtracer SV-C3200 machine (Mitutoyo Corporation, Kanagawa, Japan).

**Figure 4:**
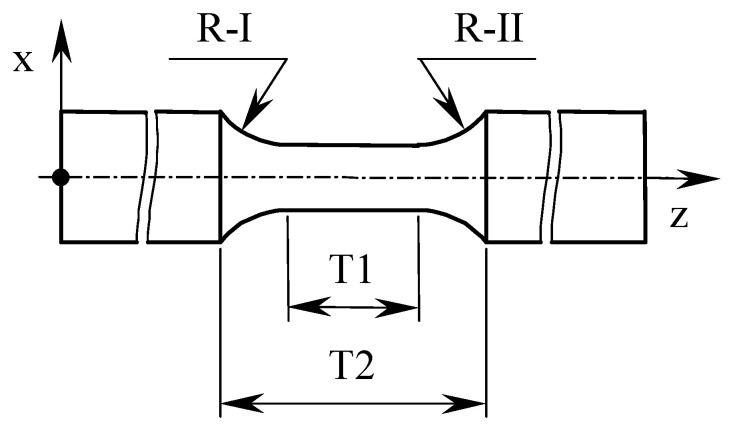
The test specimen with marked geometrical dimensions measurement points.

The test samples were assessed for accuracy in the scope of the required shape in accordance with [26] standard. Ten samples were estimated. The measurements concerned the determination of selected geometrical dimensions, i.e., the values of the R-I and R-II offset radius and the T1 working part length, as well as the gripping parts distance T2 of the sample (Figure 4). 

Table 2 gives the geometric measurements results for the DB and for the DMLS samples: offset radius values R-I and R-II, measuring part T1 and T2 length, according to Figure 4. The obtained results were presented in statistical terms using the following parameters: mean value, standard deviation, minimum value, and maximum value.

The most important dimensions of cylindrical samples are the offset radius values, which determine the value of the stress concentration factor affecting the test objects damage form. The lack of geometric features repeatability may affect the value of the determined Ti6Al4V mechanical parameters. The measurements results presented in Table 2 were carried out for 10 samples. Samples were tested in the same manner that was consistent with their production method.

The offset radius measurements of turned DB samples indicate that they are characterized by dimensional repeatability. The difference between the average R-I and R-II radius is 0.007 mm, which should be considered as insignificant. Analyzing the minimum value of R-I and R-II radius, the difference in value is 0.098 mm, which should be considered as insignificant. Taking into consideration the maximum value of R-I and R-II radius, the difference in value is 0.914 mm, which should be considered a significant difference. The difference in the value of R-I (calculated as the difference between the maximum and minimum values) is 0.234 mm, while the difference in the value of the R-II radius is 1.05 mm.

The offset radius measurements of samples produced using the DMLS method indicate that they are characterized by dimensional repeatability. The difference between the average R-I and R-II radius is 0.136 mm, which should be considered as insignificant. The difference in minimum value of R-I and R-II is 0.136 mm, which should be considered as insignificant. Analyzing the maximum value of R-I and R-II, it can be seen that the difference in value is 0.162 mm, which should be considered as insignificant. The difference in the value of R-I (calculated as the difference between the maximum and minimum values) is 0.632 mm, while the difference in the value of the R-II radius is 0.606 mm. Samples made using the additive method are characterized by repeatability of geometric features, which is important in the context of repeatability of the strength tests results. 

#### 2.3.2. Working Part Diameter Measurement

For the test part of the specimen, the diameter value was measured, and the roundness was determined. The measurements were carried out with the Mitutoyo CRYSTA-APEX S574 device (Mitutoyo Corporation, Kanagawa, Japan).

**Figure 5:**
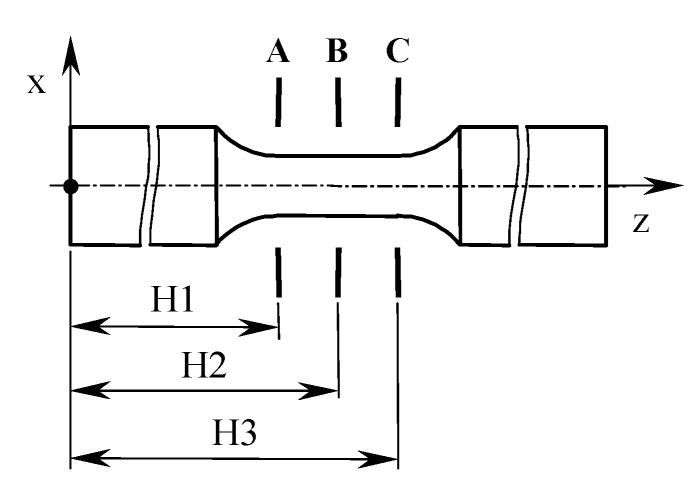
Designation of the cross-sections in which the diameter values were measured. The distance of the given cross-section (A, B, C) from the sample base was: H1 = 40 mm, H2 = 43 mm, H3 = 46 mm.

The measurements were carried out in three sections in accordance with Figure 5.

Table 3 presents the measuring results of the diameter and roundness values using the following statistical parameters: mean value, standard deviation, minimum value, and maximum value. The listed parameters were determined on the basis of 10 samples’ test results in three sections. 

**Figure 6:**
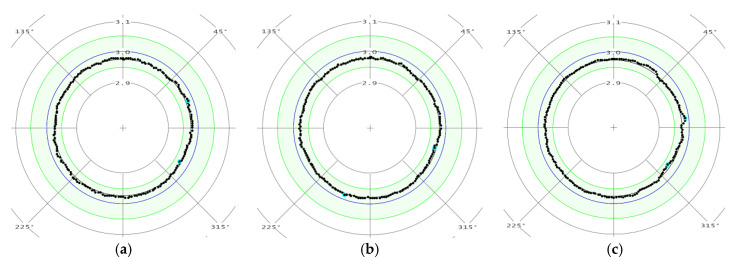
The measurements results example of the roundness of a specimen made using the DMLS method in cross-section: (**a**) A-A; (**b**) B-B; and (**c**) C-C.

**Figure 7:**
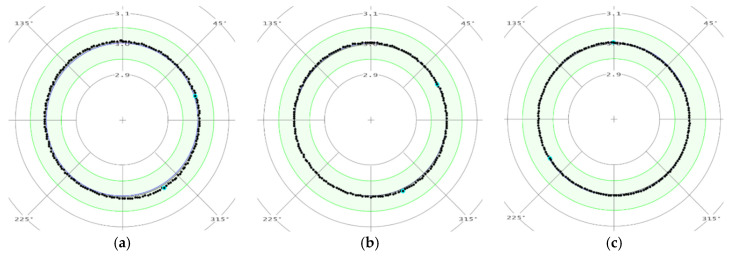
The measurements results example of the roundness of a turned DB specimen in cross-section: (**a**) A-A; (**b**) B-B; and (**c**) C-C.

The measuring results of the roundness in selected sections (A-A, B-B, and C-C) for a sample made using the DMLS method are shown in Figure 6, and for a turned DB sample are presented in Figure 7. In Figure 6 and Figure 7, elementary measurements of individual sections are illustrated with black spots. They are in the green field indicating the range of the roundness tolerance field. The blue line represents the specimen cross section nominal size equal to 6 mm.

The diameter measurements of the working part showed that samples made using the additive method (DMLS specimen) and turned samples from a drawn bar (DB specimen) are within the dimensional tolerance (±0.05 mm) established according to [27] standard. 

Analysis of the measuring points distribution for DMLS samples’ individual cross-sections regarding the roundness measurement indicates an irregular shape in relation to the nominal circle. This confirms the distribution of points shown in Figure 6c. In the case of DB specimen, the roundness measurement results show shape regularity in the examined cross-sections.

#### 2.3.3. Roughness Measurement of the Specimen Working Part

Surface roughness tests of the specimen measuring part were carried out using the Mahr MarSurf GD 120 device (GmbH, Göttingen, Germany). 

**Figure 8:**
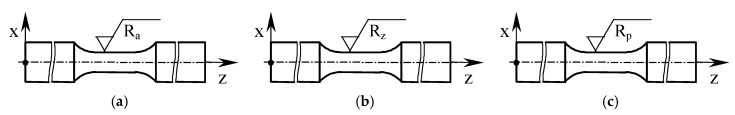
Specimens with the marking points of roughness measurement for: (**a**) R_a_ parameter; (**b**) R_z_ parameter and (**c**) R_p_ parameter.

In accordance with the recommendations and requirements of [28] standard, the following roughness parameters were determined: R_a_, R_z_, and R_p_. The tests were carried out on the measuring part of the sample, as shown in Figure 8.

R_a_ specifies the arithmetic mean profile deviations from the average line measured along the measuring section. R_z_ is the arithmetic mean value of the absolute heights of the five highest profile elevations and the five lowest dimples on the elementary segment. R_p_ is the height of the highest profile peak. 

The indicated roughness parameters values were determined on the basis of the same measuring sections. The obtained test results are presented in Table 4.

The test results analysis indicates that the average value of all roughness parameters (i.e., R_a_, R_z_, and R_p_) is higher for DMLS specimens than the values obtained for DB specimens. The differences result from the fact that the DMLS samples were not subjected to additional mechanical treatment after their production. This form of samples was adopted for testing because complex construction elements made using the DMLS method are not subjected to additional mechanical treatment. The percentages difference of the DMLS specimens’ average roughness parameters in relation to DB samples are: for R_a_. 690.2%; for R_z_. 769.4%; for R_p_. 693.8%. The maximum values analysis of selected roughness parameters allowed determining their percentage differences: for R_a_. 511.0%; for R_z_. 667.3%; and for R_p_. 636.8%. Analyzing the minimum values of selected parameters, their percentage differences were determined as: for R_a_. 998.1%; for R_z_. 882.3%; and for R_p_. 744.4%. The analysis showed that the R_p_ parameter best describes the profile shape variability of the specimen measuring part. It results from the percentage differences of the indicated parameter, which changed within the smallest ranges of average, minimum, and maximum values.

### 2.4. Hardness Measurement

The Vickers method was adopted to measure hardness in samples turning from a bar and printed using the DMLS additive manufacturing technology. This process involves pressing a diamond pyramid with a square base and an angle between the opposite walls of 136° into the metal, under load F during time t. The hardness parameter is the load ratio to the side surface durable imprint.

The hardness measurement was carried out in accordance with the [29] standard. The test stand was equipped with a HUATEC Vickers HV-10 hardness tester (Huatec Group Corporation, Beijing, China). Hardness tests were carried out on three samples.

During the tests, an indenter was used in the form of a four-sided shaped diamond pyramid with an apex angle of 136°. The measuring load was about 98.07 N, which allowed determining the hardness on the HV10 scale.

**Figure 9:**
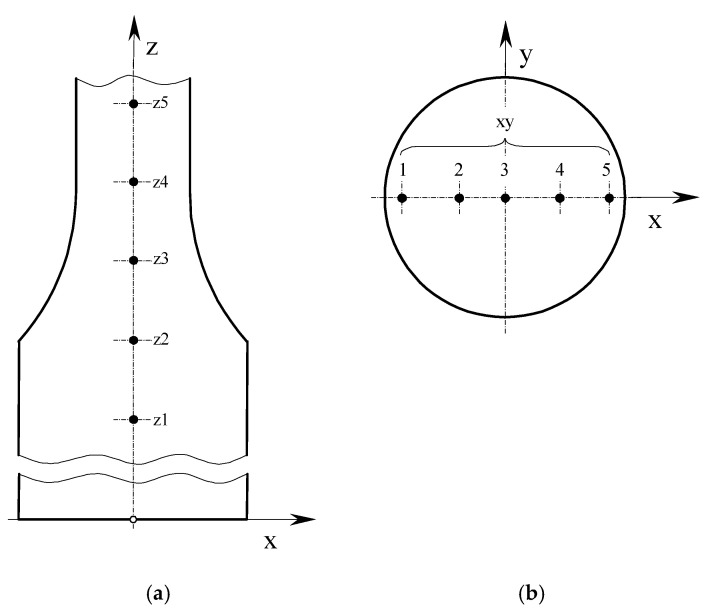
Hardness measurement method: (**a**) schematic designation of hardness measuring points along the z-axis; and (**b**) schematic designation of hardness measurement points in the shank on the x–y-plane.

Hardness measurements were carried out on metallographic sections taken from broken tensile specimens made by the DMLS method and from a drawn bar by the turning process. The material hardness was tested at five points in two planes according to the diagram in Figure 9a,b.

Based on the measurements carried out, the following hardness results were obtained (Table 5). Columns 3 and 4 of Table 5 present the hardness measurements results along the *z*-axis (Figure 9a) for the DMLS and DB specimens. Columns 6 and 7 of Table 5 contain the results achieved on the x-plane of the DMLS and DB samples gripping part (Figure 9b).

### 2.5. Material Strength Tests

To determine the mechanical properties of the Ti6Al4V material produced in the turning process (from a drawn bar) and by the DMLS additive manufacturing technology, a static tensile test was performed in accordance with the [24] standard.

Material strength tests were carried out on three samples. The stand for determining the strength parameters was an Instron 8502 hydraulic machine (Instron, Norwood, MA, USA). The control parameter during the tests was a machine piston displacement of 0.05 mm/s. The loading force and deformation were recorded during the tests. The tests were accomplished using an extensometer (Instron, Norwood, MA, USA) with a measuring base of 10 mm and a range of 1 mm. 

During the static tensile test, the strain value was measured in two ways. The first method was based on the use of an extensometer mounted directly on the sample. The second way to measure sample deformation was the digital image correlation method. This method consisted in measuring and monitoring the displacements of the specimen observed fragment, which was in the view field of the BASLER acA4024-8gm camera (Basler AG, Ahrensburg, Germany) set on a tripod on a solid surface.

The DIC method measurement consisted of an images series periodically recorded over a specified period of time. Usually, the first image was the reference image [30]. The method adopted was to photograph the sample during a tensile test with a camera at constant intervals. The images recorded during the research were subjected to displacement analysis using BASLER’s Pylon software (version 6.1.0, Basler AG, Ahrensburg, Germany). This program compared all the photos recorded during the test to the reference image. It was based on determining the position of a given pixel on the reference image and ascertaining the displacement of this point on subsequent photos. 

**Figure 10:**
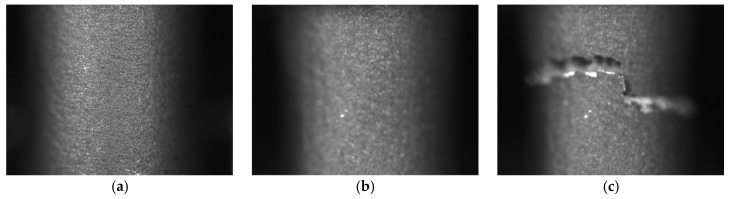
Pictures of a DMLS specimen taken with the BASLER acA4024-8gm camera during a static tensile test: (**a**) before starting the test (reference image); (**b**) just before the sample breaks and (**c**) just after breaking up.

**Figure 11:**
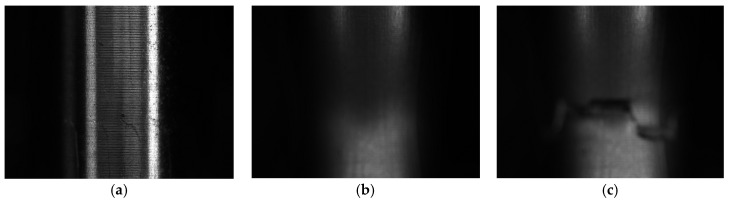
Pictures of a DB specimen produced by the turning method taken with the BASLER acA4024-8gm camera during a static tensile test: (**a**) before starting the test (reference image); (**b**) just before the sample breaks and (**c**) just after breaking up.

Figure 10 and Figure 11 show sample photos taken with the BASLER acA4024-8gm camera during a static tensile test for two types of specimens. Figure 10 shows a sample produced by DMLS technology, while Figure 11 shows a turned sample from a drawn bar. Figure 10a and Figure 11a are reference images registered first during the static tensile test. Figure 10b and Figure 11b are photos taken just before the samples were broken. Figure 10c and Figure 11c are images of already broken specimens. 

As a result of the static tensile test, the following material mechanical properties were determined: tensile strength S_u_, yield strength S_y0.2_, Young’s modulus E, elongation A, and narrowing Z.

**Figure 12:**
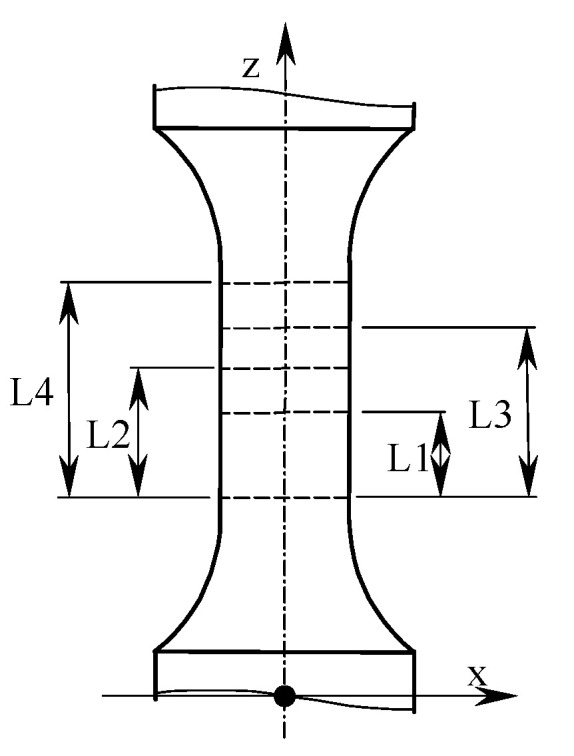
Schematic presentation of the areas analyzed using the digital image correlation method, for which the measuring ranges were: L1 = 1.36 mm, L2 = 2.72 mm, L3 = 3.49 mm, and L4 = 10 mm (measured with an extensometer).

**Figure 13:**
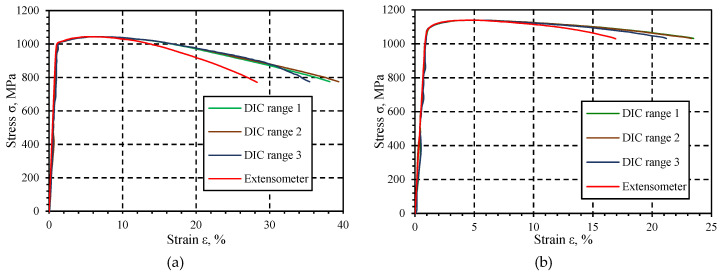
An example of the stretching graph S = f(ε) Ti6Al4V titanium alloy: (**a**) DMLS specimen and (**b**) DB specimen.

Figure 12 schematically shows the area on the sample subject to displacement analysis, which was divided into three ranges. The selected strength parameter values obtained for DMLS samples are presented in Table 6, while the results for specimens produced of drawn bar are given in Table 7. Table 6 and Table 7 summarize the values obtained on the basis of deformation measurements made with an extensometer and using digital image correlation for various measuring ranges. The extensometer measuring range was L4 = 10 mm, while the measuring ranges for the digital image correlation method were: L1 = 1.36 mm, L2 = 2.72 mm, and L3 = 3.49 mm. Figure 13 shows examples of tensile charts determined using an extensometer and digital image correlation for samples made with DMLS additive manufacturing technology (Figure 13a) and from a drawn bar by turning (Figure 13b).

The data obtained present that the strength parameters, such as yield point S_y0.2_, tensile strength S_u_, and elongation, achieved for the extensometer have lower values compared to the digital image correlation method results. The tensile tests using the extensometer for DMLS and DB samples showed a difference in Young’s modulus values. They result from different material production technologies. Comparing the results of the Young’s module for DMLS samples, the values obtained for different measurement bases of the DIC method are similar. This indicates an even deformation of the layers that resulted from the element production by the DMLS method. The differences in the yield point values are small and within the limits of measurement uncertainty. For DB samples, significant differences were observed in the Young’s modulus values for different measurement bases of the DIC method. It is presumed that the results achieved are related to locally changing properties of the sample. It is also assumed that the DIC measurement method may have influenced the results of the Young’s module determined for DB samples. This method is based on images of the sample’s surface. The small surface roughness could affect the readings of the displacement values, which contributed to obtaining specific test results. The modulus of elasticity E obtains the highest value for the extensometer. Based on the results, the following relationship is observed: the larger is the measurement area, the greater are the Young’s modulus E and elongation A, while the lower is the yield point S_y0.2_. The yield point S_y0.2_ value is close to the value obtained for the extensometer. The results obtained for turned samples behave in a similar way, as given in Table 7 and Figure 13b. The only difference is that for range 2 the highest value of elongation A was obtained.

The strength tests showed that the samples produced with the DMLS additive manufacturing technology are characterized by higher tensile strength S_u_ and yield point S_y0.2_ compared to turned DB specimens. Similar values of Young’s modulus were achieved for both sample technologies. The elongation A average value reaches almost two times higher value for specimens made of drawn bar in the turning process.

## 3. Analysis of Test Results

### 3.1. Analysis of Sample Geometric Features

Analyzing roughness measurements, it can be seen that samples made using the DMLS technology immediately after the printing process do not meet the requirements of the [27] standard. It gives the parameter Ra value of the specimen working part equal to 0.32 μm. Due to the performance of tests under static loads, the surface roughness value is not a factor that significantly affects the test results. Considering the results of specimens’ geometric measurements, it should be noted that they do not all meet normative assumptions in as-built state. The surface roughness Ra average value of the measuring part for the tested samples is higher than that given in the standard. On the other hand, the diameters and roundness deviations of the working part meet the normative conditions. To ensure compliance of geometric dimensions, shape, and surface roughness, additional mechanical treatment should be carried out.

### 3.2. Hardness Test Results Comparison

**Figure 14:**
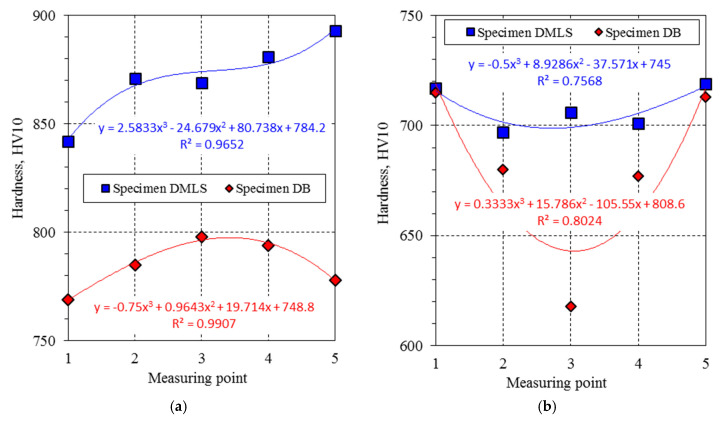
HV10 hardness distribution: (**a**) along the z-axis; and (**b**) in the shank on the x–y-plane.

On the basis of the hardness tests, a difference in the results obtained for samples produced from a drawn bar and made using the additive manufacturing method was found. According to Figure 9a, five hardness measurements of DMLS and DB specimens were made along the z-axis. Analyzing the achieved hardness values presented in Figure 14a, it can be seen that, for the DMLS specimen, the hardness along the *z*-axis is higher than the hardness for the DB sample. For the DMLS specimen, the hardness results are in the range 842–893 HV10, while the hardness for the DB specimen varies between 769 and 798 HV10. According to the measurement implementation scheme (Figure 9b), the first measuring point is located in the sample gripping area, while the last measuring point is located closest to the edge of the sample crack. For the DMLS sample, it can be considered that the hardness on the *z*-axis increases with the approach to the crack site. The highest hardness values for the DMLS specimen (893 HV10) were obtained near the fracture site of the sample, while, for the DB specimen, the highest hardness (798 HV10) was achieved at the transition point between the shank and measuring part. The hardness at the first point located in the gripping part for the DMLS specimen is 842 HV10 and for the DB specimen 769 HV10. The increase in hardness at the DMLS fracture site may be related to the deformations occurring in this area caused by the static tensile test. The increase in hardness at the DMLS fracture site may be related to the deformations occurring in this area caused by the static tensile test. This is due to the change in the sample geometry, which affects the change in stress/strain values in specific sections. The highest hardness occurs in the area where the crack occurred. This is where the highest deformations occurred, which were close to 16.9%. In the case of a sample made of drawn bar, the change in the hardness value along the *z*-axis is related to the higher ductility of this material compared to the DMLS sample. In the gripping part of the sample, the rising of deformations caused by the static tensile test increases the hardness of the material. This is due to the change in the value of the sample cross-sections. On the other hand, a significant increase in these deformations in the measuring part of the sample causes significant changes in the structure of the material contributing to a decrease in its hardness. In addition, Figure 14a presents the measurement results as a polynomial function whose equation was determined for two types of samples. In each case, the determination coefficient value was R^2^ > 0.95, which indicates a good fit of the polynomial function with the experimental research results.

Figure 14b shows the hardness measuring results of the DMLS and DB samples taken on the x–y-plane according to the diagram in Figure 9b. The hardness results for both DMLS and DB specimens were lower than those achieved along the z-axis. On the x-plane, higher hardness values were gained for the DMLS specimen, which were in the range of 697–719 HV10. On the other hand, the DB sample had a hardness of 618–715 HV10. The DB sample was characterized by the highest hardness around the section edge Point 1, 715 HV10; Point 5, 713 HV10). As the DB sample axis approaches, a decrease in hardness can be seen (Point 3, 618 HV10). Such hardness distribution may be affected by the deformations value resulting from the stretching of the material outer layers. It is known that a rod with a round section loaded with tensile force has a variable distribution of deformations in the cross section. Therefore, in each of the analyzed cases, the hardness outside the sample is the highest. In the case of DB samples, this difference may be due to the producing method of the bar, i.e., pulling process. The obtained results were described by the polynomial equation shown in Figure 14b, which does not describe their variability very accurately, as evidenced by the determination coefficient R^2^ = 0.8024. In the case of results for DMLS specimen, the highest values were around the section edge (Point 1, 717 HV10; Point 5, 719 HV10). As with the DB sample, the hardness value was the highest on the outer layers of the sample. It can also be affected by deformations occurring in the material resulting from the stretching of the outer layers. Differences in hardness values in the printed sample were lower due to the layered manufacturing process. The difference between the highest and lowest hardness values for the DMLS sample was 22 HV10. The obtained test results were described by a polynomial equation (Figure 14b). The value of the coefficient of determination is R^2^ = 0.7568.

### 3.3. Analysis of Static Tension Test Results

**Figure 15:**
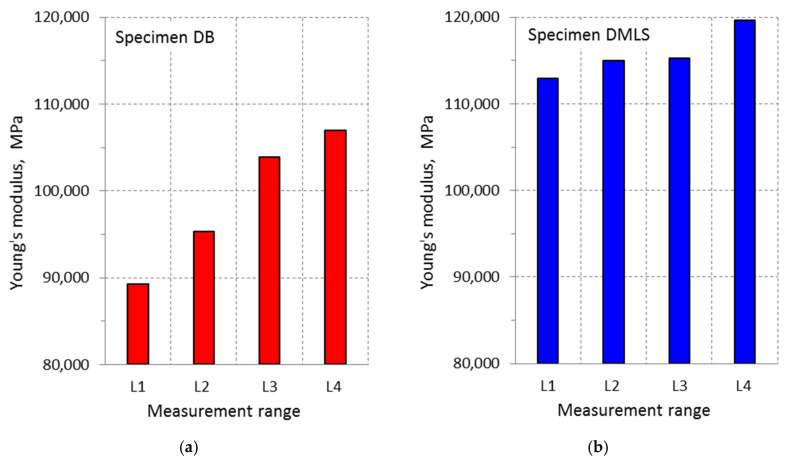
Change of Young’s modulus value depending on changes in the measurement base value for: (**a**) DB specimen; and (**b**) DMLS specimen.

The tests carried out on titanium alloy Ti6Al4V allowed determining the yield point value S_y0.2_ and tensile strength S_u_ for DB and DMLS specimens. The indicated parameters have higher values for DMLS samples (S_u_ = 1140 MPa) in relation to DB samples (S_u_ = 1044 MPa). In the case of DB samples for the measuring ranges variable value, the quotient was S_y0.2_/S_u_ ≈ 1.04, while for DMLS specimen its value was S_y0.2_/S_u_ = 1.03 ± 1.05. Analyzing the Young’s modulus values as a function of the variable measuring range value, it was found that, in the case of DB specimens, the lowest module values E = 89,234 MPa were obtained for the range L3 = 3.49 mm, while the highest module values E = 106,940 MPa for the range L4 = 10 mm. The difference in values is ΔE = 17,706 MPa (Figure 15a). In the case of DMLS samples, the lowest module values E = 112,950 MPa were gained for the range L3 = 3.49 mm, while the highest module values E = 119,610 MPa for the range L4 = 10 mm. The difference in values is ΔE = 6660 MPa (Figure 15b). The DIC method is ideal for determining local mechanical properties that may be used, e.g., in numerical calculations of construction elements. Based on the tests carried out, it can be stated that, for a Young’s modulus, the difference in the parameter E value depends on the measurement base: for samples made of drawn rod, it was about 14%, while, for samples made by the additive method, it was about 2%.

In the case of DMLS samples:

- for measuring base L1 = 1.36 mm, module E = 115,300 MPa;
- for measuring base L2 = 2.72 mm, module E = 115,020 MPa; and
- for measuring bases L3 = 3.49 mm, module E = 112,950 MPa.

Similar Young’s modulus values for DMLS samples result from sample-making technology. The specimen was made along the z-axis, which indicates that the subsequent layers’ connections of the element were tested. A small measurement base adoption reduced the number of analyzed material layers, and thus the material defects number occurring in the analyzed volume. Material defects may result from: no melting of the powder, occurring microporosity, material defects, etc.

Young’s modulus is a material constant, thus its value should be the same for each of the measurement methods. Changes in the values of Young’s modulus for individual measuring ranges in the DMLS sample may be related to local changes in material properties. The individual measurement bases include small fragments of the sample in the area of the greatest deformation and subsequent cracking. As a result, the greatest local changes in material properties occur in this area. The difference in the Young’s modulus values resulted from the inaccuracy of the digital image correlation method. Analyzing the test results, it can be concluded that the DIC method is not reliable compared to the standard method using an extensometer used in engineering research. In the case of both DB and DMLS samples, the value of Young’s modulus E decreased with the increase of the L area under analysis. This may result from poor lighting of the sample during the tests (too dark or overexposed photos), which translates into “losing” points by the program during the analysis. The differences between Young’s modulus obtained for the measurement ranges L1, L2, and L3 for the DMLS sample are smaller than for the DB sample. The surface of the DMLS sample was rougher and dull, which favored taking higher quality photos, while the turned DB sample had a shiny surface, which reflected the light, causing the photos to be overexposed. As a result of the conducted research, it can be concluded that the DIC method depends on many factors, i.e., the surface of the tested element and its preparation, appropriate lighting, and the size of the analyzed area. It was found that the DIC method can be reliable for small measurement areas. Comparing the results obtained using the DIC method and the results achieved using an extensometer, it was found that they differ in the range of about 5%.

**Figure 16:**
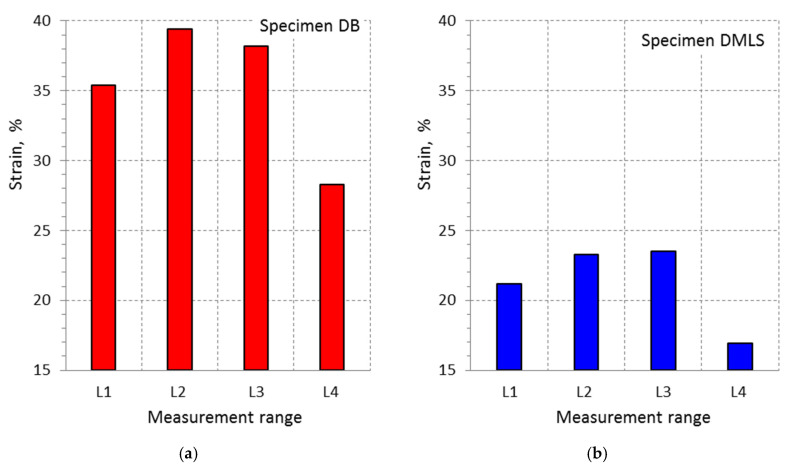
Change in the samples elongation depending on changes in the value of the measurement base for: (**a**) DB specimen; and (**b**) DMLS specimen.

The changes results in elongation A for the adopted measuring ranges show that the lowest values were achieved for both types of samples for L4 = 10 mm (Figure 16). The smaller the measurement range, the greater the strain value. The smallest measuring range included a fragment of the sample in which there was a crack resulting from the occurrence of tensile forces. There was stress concentration at the crack site and a simultaneous increase in the deformation of the material layers. The average deformation value in areas L2 and L3 was lower than in the case of the L1 measurement range. The largest measuring range L4, obtained for the extensometer, was characterized by the lowest deformation value. Higher elongation values were obtained for DB specimens that range from 28.3% to 39.4%, while for DMLS samples from 16.9% to 23.5%.

## 4. Conclusions

DIC is an optical method that allows measuring displacements that can be used to determine strains caused by external loads. In the case of material produced by the additive method, based on sintering of metallic powder layers, its strength is determined by the strength of the individual layer connections. The DIC method is ideal for determining local mechanical properties that may be used, e.g., in numerical calculations of construction elements. Based on the tests carried out, it can be stated that, for a Young’s modulus, the difference in the parameter E value depends on the measurement base: for samples made of drawn rod, it is about 14%, while, for samples made by the additive method, it is about 2%.

Analysis of changes in the Young’s modulus value showed that the highest values were obtained for the measuring range L4 = 10 mm for both types of specimens. Young’s modulus for DMLS samples was 11.8% larger than module E for DB samples. The variability of the module E value for the adopted measuring ranges was much lower for DMLS specimens. It is related to the technology of sample preparation by the DMLS additive manufacturing method consisting in sintering material layers. Therefore, the structural element strength is determined by the strength of a single joint of the material in two layers.

Ti6Al4V titanium alloy produced from a powder by the DMLS additive manufacturing method showed higher values of Young’s modulus E in relation to the results achieved for the drawn bar material.

The assumed strain measuring ranges importantly influenced the value of Young’s modulus for specimens made of drawn bar (ΔE = 17,706 MPa), while, in the case of DMLS samples, this impact was significantly smaller (ΔE = 6660 MPa). The experimentally determined Young’s modulus value is important for calculations performed using numerical methods.

The hardness of specimens produced using the DMLS method was higher than in the case of samples made from a drawn bar by turning. This applied to hardness along the *z*-axis and on the x–y-plane.

Producing samples using the DMLS additive technology allowed obtaining surface roughness characterized by average values: R_a_ = 1.898 mm, R_z_ = 11.880 mm, and R_p_ = 5.585 mm.

Comparison of the obtained results with those presented in [6,7,8,9,10] indicates that they are consistent with the results achieved for the annealed Ti6Al4V material. High results compliance was obtained with the results presented in this work for all analyzed parameters, i.e., S_y_, S_u_, E, and A.

## Figures and Tables

**Table 1:** Chemical composition of the Ti6Al4V titanium alloy according to [11].

Main Alloying Elements and Their Content in the Alloy Ti6Al4V, %							
Al	V	Fe	O	N	C	H	Ti
5.5 ÷ 6.75	3.5 ÷ 4.5	≤0.3	≤0.2	≤0.05	≤0.08	≤0.01	rest

**Table 2:** Geometric measurement results of Ti6Al4V samples produced from a drawn bar (DB specimen) and by the DMLS method (DMLS specimen).

	Selected Geometrical Parameters							
	Geometry of Turned DB Specimens				Geometry of the DMLS Specimens			
	R-I	R-II	T1	T2	R-I	R-II	T1	T2
	mm	mm	mm	mm	mm	mm	mm	mm
Mean Value	8.016	8.090	12.834	24.996	8.077	7.941	12.677	24.339
Standard Deviation	0.071	0.316	0.354	0.058	0.183	0.176	0.131	0.103
Minimum Value	7.835	7.933	11.957	24.935	7.779	7.643	12.424	24.259
Maximum Value	8.069	8.983	13.161	25.099	8.411	8.249	12.915	24.590

**Table 3:** Measurement results for the roundness and diameter deviations of the specimen measuring part.

	The Specimen Measuring Part Geometric Parameters			
	Specimen DB		Specimen DMLS	
	Diameter	Roundness	Diameter	Roundness
	mm	mm	mm	mm
Mean Value	5.994	0.009	5.949	0.019
Standard Deviation	0.007	0.004	0.004	0.004
Minimum Value	5.984	0.003	5.942	0.013
Maximum Value	6.012	0.015	5.958	0.027

**Table 4:** Surface roughness measurements results for the measuring part of the specimen.

	Specimen Roughness Parameters					
	Specimen DB			Specimen DMLS		
	R_a_	R_z_	R_p_	R_a_	R_z_	R_p_
	μm	μm	μm	mm	μm	μm
Mean Value	0.275	1.544	0.805	1.898	11.880	5.585
Standard Deviation	0.089	0.385	0.181	0.182	1.339	0.633
Minimum Value	0.161	1.132	0.617	1.607	9.988	4.593
Maximum Value	0.428	2.096	1.120	2.187	13.987	7.132

**Table 5:** Vickers hardness measurement results.

No.	Vickers Hardness Measurement Method (HV10)					
	Along z Axis			On the x–y Plane		
	No	Specimen DMLS	Specimen DB	No	Specimen DMLS	Specimen DB
1	z1	842	769	xy1	717	715
2	z2	871	785	xy2	697	680
3	z3	869	798	xy3	706	618
4	z4	881	794	xy4	701	677
5	z5	893	778	xy5	719	713

**Table 6:** Selected strength parameters list of the Ti6Al4V titanium alloy manufactured by the DMLS method under static tensile loads.

	Measurement Range	Selected Strength Parameters Average Values				
		S_y0.2_	S_u_	E	A	Z
		MPa	MPa	MPa	%	%
DIC Range 1	L1 = 1.36 mm	1089 ± 40	1140 ± 20	115,300 ± 2037	23.5 ± 3.3	19.2 ± 3.5
DIC Range 2	L2 = 2.72 mm	1091 ± 35	1140 ± 31	115,020 ± 2805	23.3 ± 2.9	19.2 ± 3.5
DIC Range 3	L3 = 3.49 mm	1096 ± 42	1140 ± 24	112,950 ± 2350	21.2 ± 3.5	19.2 ± 3.5
Extensometer	L4 = 10 mm	1086 ± 28	1121 ± 42	119,610 ± 1254	16.9 ± 4.3	19.2 ± 3.5

**Table 7:** Selected strength parameters list of the Ti6Al4V titanium alloy in the drawn bar form under static tensile loads.

	Measurement Range	Selected Strength Parameters Average Values				
		S_y0.2_	S_u_	E	A	Z
		MPa	MPa	MPa	%	%
DIC Range 1	L1 = 1.36 mm	1006 ± 55	1044 ± 48	103,880 ± 4709	38.2 ± 0.9	47.3 ± 6.2
DIC Range 2	L2 = 2.72 mm	1008 ± 47	1044 ± 29	95,316 ± 3897	39.4 ± 1.3	47.3 ± 6.2
DIC Range 3	L3 = 3.49 mm	1008 ± 31	1044 ± 46	89,234 ± 3564	35.4 ± 1.8	47.3 ± 6.2
Extensometer	L4 = 10 mm	1004 ± 34	1042 ± 37	106,940 ± 4375	28.3 ± 2.2	47.3 ± 6.2
